# Association between Decreased SGK1 and Increased Intestinal α-Synuclein in an MPTP Mouse Model of Parkinson’s Disease

**DOI:** 10.3390/ijms242216408

**Published:** 2023-11-16

**Authors:** Min Hyung Seo, Dasom Kwon, Soo-Hwan Kim, Sujung Yeo

**Affiliations:** 1 Department of Meridian and Acupoint, College of Korean Medicine, Sang Ji University, Wonju 26339, Republic of Korea; cstcl@naver.com (M.H.S.); kdsom618@naver.com (D.K.); 2 Division of Biological Science and Technology, Yonsei University, 1 Yonseidae-gil, Wonju 26493, Republic of Korea; 3 Research Institute of Korean Medicine, Sangji University, Wonju 26339, Republic of Korea

**Keywords:** Parkinson’s disease, SGK1, α-synuclein, Na^+^/K^+^ pump ATPase, SW480

## Abstract

Parkinson’s disease (PD) is a globally common progressive neurodegenerative disease resulting from the loss of dopaminergic neurons in the brain. Increased α-synuclein (α-syn) is associated with the degeneration of dopaminergic neurons and non-motor symptoms like gastrointestinal disorders. In this study, we investigated the association between serum/glucocorticoid-related kinase 1 (SGK1) and α-syn in the colon of a PD mouse model. SGK1 and α-syn expression patterns were opposite in the surrounding colon tissue, with decreased SGK1 expression and increased α-syn expression in the PD group. Immunofluorescence analyses revealed the colocation of SGK1 and α-syn; the PD group demonstrated weaker SGK1 expression and stronger α-syn expression than the control group. Immunoblotting analysis showed that Na^+^/K^+^ pump ATPase α1 expression levels were significantly increased in the PD group. In SW480 cells with SGK1 knockdown using SGK1 siRNA, decreasing SGK1 levels corresponded with significant increases in the expression levels of α-syn and ATPase α1. These results suggest that SGK1 significantly regulates Na^+^/K^+^ pump ATPase, influencing the relationship between electrolyte balance and fecal formation in the PD mouse model. Gastrointestinal disorders are some of the major prodromal symptoms of PD. Therefore, modulating SGK1 expression could be an important strategy for controlling PD.

## 1. Introduction

Parkinson’s disease (PD), first reported by James Parkinson in 1871 [1,2], is the second most common progressive neurodegenerative disease globally. Although it used to be a rare condition, its prevalence doubled from 1990 to 2015, affecting over six million people. It is projected that over 12 million people will have PD by 2040. PD is characterized by motor symptoms, such as bradykinesia, tremor, and rigidity, resulting from reduced dopaminergic neurons in the substantia nigra (SN) and non-motor symptoms, such as gastrointestinal disorders (constipation, etc.) [3,4,5,6,7]. Although the exact causes and mechanisms of PD are unclear, factors such as misfolding and accumulation of the synaptic protein α-synuclein (α-syn) are reportedly associated with the degeneration of dopaminergic neurons [8,9]. Non-motor symptoms, such as gastrointestinal disorders, have also been associated with increased intestinal α-syn [10,11,12]. The elevated levels of α-syn in the intestines are likely to play a pivotal role in the underlying pathology of PD [13,14]. Increased intestinal α-syn expression has not been documented in Alzheimer’s disease, and PD reportedly occurs selectively in a subset of the intestinal α-syn-positive population or may induce this expression [15]. Moreover, increased α-syn migrates to the brain and influences the pathology of PD [9,13,14,16]. This hypothesis of a gut–brain axis in PD was put forward by Braak et al. in 2003; they presented a staging system for PD based on α-syn deposition patterns identified in cases of autopsy [12,17].

Serum/glucocorticoid-related kinase 1 (SGK1), known for its activation by insulin and growth factors, is an enzyme that participates in various physiological and pathological processes [18]. Its functions include regulating cell survival, growth, differentiation, inflammation, electrolyte balance, and metabolism [18,19,20,21]. It is also involved in the pathophysiology of several diseases, including PD. Under oxidative stress, SGK1 exhibits a protective role [19,20,22]. Oxidative stress is a possible cause of neurodegenerative diseases, such as PD. The overexpression of SGK1 prevents cell death; therefore, SGK1 can overcome this oxidative stress [23,24,25,26].

Our previous study indicated that reduced SGK1 expression may significantly contribute to the upregulation of α-syn and may be associated with the death of dopaminergic cells in the substantia nigra (SN) and the muscles of mice with chronic Parkinsonism [27,28]. Gastrointestinal disorders, especially constipation, are major prodromal symptoms of PD [3,5]. Therefore, investigating the association between α-syn and elements of gastrointestinal disorders is important. SGK1 is related to Na^+^/K^+^ pump ATPase, and intestinal Na^+^/K^+^ pump ATPase α1 and ß1 have been reported to be present [29,30].

In this study, based on the report that 1-methyl 4-phenyl 1,2,3,6-tetrahydropyridine (MPTP) is a representative dopamine neuron toxin of the enteric nervous system inducing gastrointestinal dysfunction [6], the association between SGK1, a powerful stimulator of Na^+^/K^+^ pump ATPase, and α-syn was examined in the colon of an MPTP-induced PD model. This study hypothesized that non-motor symptoms of PD, such as constipation [7], could be caused by changes in SGK1 expression related to Na^+^/K^+^ pump and α-syn expression changes. A close association was reported between the basolateral Na^+^-K^+^-2Cl^-^ cotransporter and intestinal water and mucus secretion, as well as its impact on the immune system [31]. As Alcian blue selectively binds to acidic mucopolysaccharides and mucins, Alcian blue staining was utilized in this study to confirm changes in mucus secretion in the colon of mice with MPTP-induced PD in response to alterations in fluid and electrolyte balance. In light of the changes in SGK1, alterations in α-syn and Na^+^/K^+^ pump-related factors were also studied in SW480 cells in this study.

## 2. Results

### 2.1. MPTP-Induced PD Model

**Figure 1:**
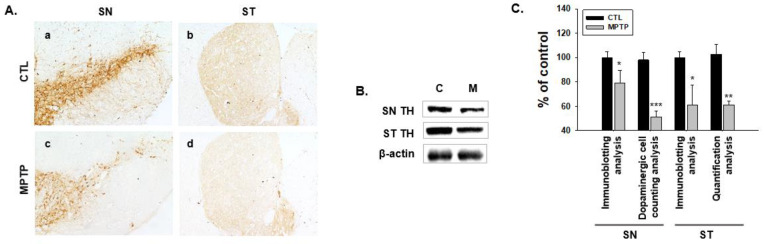
Changes in the expression patterns of tyrosine hydroxylase (TH) in the substantia nigra (SN) and striatum (ST) regions of the brain in 1-methyl-4-phenyl-1,2,3,6-tetrahydropyridine (MPTP)-induced Parkinson’s disease model. (**A**) Immunohistochemistry analyses showing that TH expression decreased in SN and ST regions in the MPTP group (**c**,**d**) compared with that in the control (CTL) group (**a**,**b**). a and c panels; 100×, b and d panels; 40×. (**B**) Immunoblotting analyses of TH in SN and ST regions in the brain. TH expression significantly decreased in SN and ST regions in the MPTP group. (**C**) Graphs indicating the results of the immunoblotting analysis, dopaminergic cell counting analysis in SN, and TH expression quantification per area in ST. CTL, intraperitoneally injected with 100 μL 0.9% saline daily for 4 weeks; MPTP, intraperitoneally injected with MPTP-HCI (20 mg/kg) daily for 4 weeks (*n* = 3, * *p* < 0.05, ** *p* < 0.005, *** *p* < 0.0005).

The PD model induced by MPTP showed significantly decreased expression levels of tyrosine hydroxylase (TH) in the SN and striatum (ST, Figure 1A,B). The number of dopaminergic cells in the SN and the TH concentration in the ST were significantly reduced (Figure 1C). Consistently, immunoblot analysis showed markedly reduced TH expression levels in the SN and ST regions (Figure 1B,C). These results demonstrate the characteristics of PD.

### 2.2. Morphological Changes in the Colon in the MPTP-Induced PD Model

**Figure 2:**
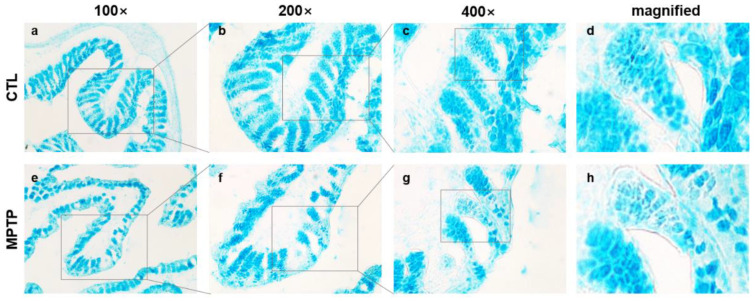
Alcian blue staining analysis in the colon of the 1-methyl-4-phenyl-1,2,3,6-tetrahydropyridine (MPTP)-induced Parkinson’s disease model. ((**a**,**e**); 100×) Staining expression patterns were changed in the MPTP group compared with those in the control (CTL) group. ((**b**,**f**); 200×) Enlarged images of staining expression pattern changes in the villi in the CTL and MPTP groups are shown. ((**c**,**g**); 400×) Enlarged images of staining expression pattern changes in the microvilli in the CTL and MPTP groups are shown. (**d**,**h**) Magnified images of panels c and g. CTL, intraperitoneally injected with 100 μL 0.9% saline daily for 4 weeks; MPTP, intraperitoneally injected with MPTP-HCI (20 mg/kg) daily for 4 weeks.

Alcian blue staining was performed to investigate the morphological changes in the colon in the MPTP-induced PD mouse model using colon tissues from the control (CTL) and MPTP-treated (MPTP) groups (Figure 2). The Alcian blue staining analyses showed that the expression of mucins was uneven in villi and microvilli of the MPTP group. The tissue matrix in the MPTP group was less sturdy than that in the CTL group. The results indicate that the density of the colon tissue significantly reduced in the MPTP group (Figure 2h).

### 2.3. SGK1 and α-Syn Expression in the Colon of MPTP-Induced PD Model

**Figure 3:**
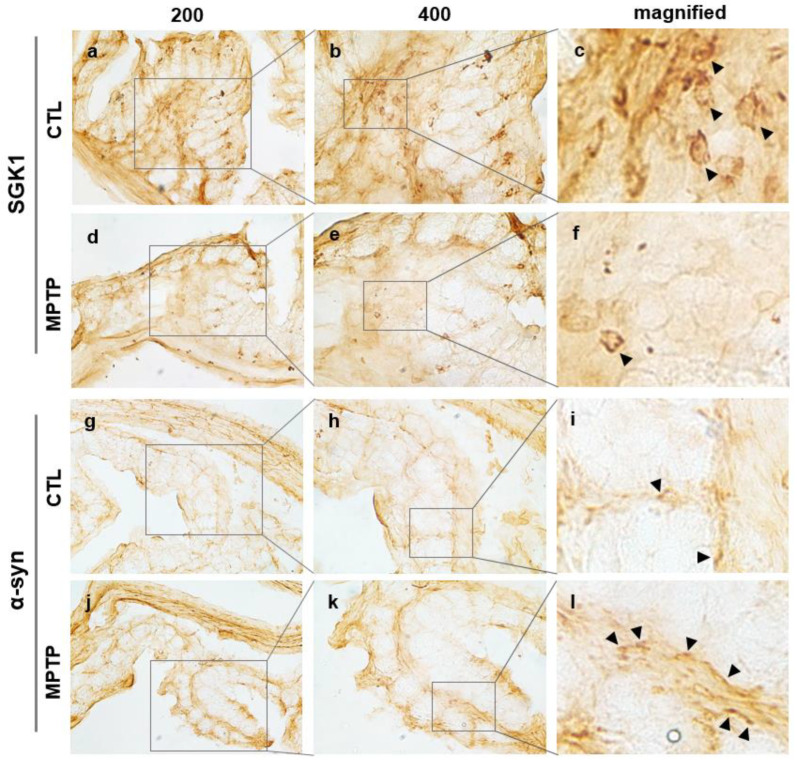
Changes in the expression pattern of serum/glucocorticoid-related kinase 1 (SGK1) and α-synuclein (α-syn) in the colon in 1-methyl-4-phenyl-1,2,3,6-tetrahydropyridine (MPTP)-induced Parkinson’s disease model. Immunohistochemistry analyses of SGK1 (**a**–**f**) showing decreased SGK1 expression in the MPTP group (**d**–**f**) compared with that in the control (CTL) group (**a**–**c**). Immunohistochemistry analyses of α-syn (**g**–**i**) showing increased α-syn expression in the MPTP group (**j**–**l**) compared with that in the control (CTL) group (**g**–**i**). SGK1 and α-syn are indicated by black arrows in panels showing magnified images (**c**,**f**,**i**,**l**). CTL, intraperitoneally injected with 100 μL 0.9% saline daily for 4 weeks; MPTP, intraperitoneally injected with MPTP-HCI (20 mg/kg) daily for 4 weeks.

Immunohistochemical analysis of SGK1 in the colon of CTL and MPTP groups was performed. To verify the report that α-syn depositions are discovered in the gastrointestinal (GI) tract, α-syn in the colon was also analyzed immunohistochemically, and the expression patterns of SGK1 and α-syn were compared in the CTL and MPTP groups (Figure 3, Supplementary Figures S1 and S2). SGK1 and α-syn expression patterns were found to be opposite in the surrounding colon tissue, with the expression level of SGK1 decreasing (Figure 3c,f) and that of α-syn increasing in the MPTP group (Figure 3i,l).

**Figure 4:**
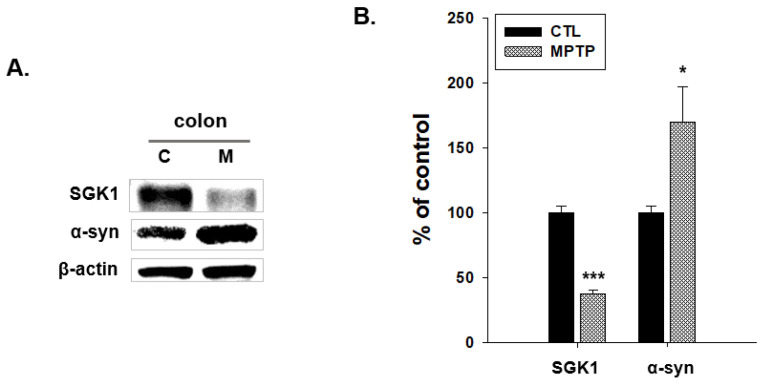
Changes in the expression pattern of serum/glucocorticoid-related kinase 1 (SGK1) and α-synuclein (α-syn) expression in the colon in 1-methyl-4-phenyl-1,2,3,6-tetrahydropyridine (MPTP)-induced Parkinson’s disease model. (**A**) Immunoblot analyses showing decreased SGK1 expression. In contrast, α-syn expression increased in the colon in the MPTP group compared with that in the control (CTL) group. (**B**) Graphs of the immunoblot analysis results. CTL, intraperitoneally injected with 100 μL 0.9% saline daily for 4 weeks; MPTP, intraperitoneally injected with MPTP-HCI (20 mg/kg) daily for 4 weeks (*n* = 3, * *p* < 0.05, *** *p* < 0.0005).

The expression patterns of SGK1 and α-syn were compared in the CTL and MPTP groups (Figure 4). The expression level of SGK1 significantly decreased, and that of α-syn significantly increased in the MPTP group compared with that in the CTL group (Figure 4B).

**Figure 5:**
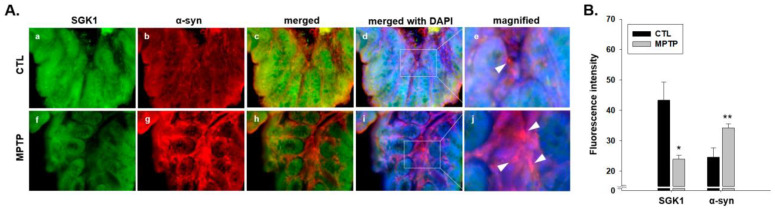
Immunofluorescence analyses of serum/glucocorticoid-related kinase 1 (SGK1) and α-synuclein (α-syn) in the colon in the 1-methyl-4-phenyl-1,2,3,6-tetrahydropyridine (MPTP)-induced Parkinson’s disease model. (**A**) Immunofluorescence analyses of SGK1 and α-syn were performed in the control (CTL) (**a**–**e**) and MPTP groups (**f**–**j**). The expression of SGK1 decreased ((**a**,**f**); 400×), whereas that of α-syn increased ((**b**,**g**); 400×) in the MPTP group. SGK1 and α-syn were merged in each group ((**c**,**h**); 400×). Nuclei were stained with DAPI ((**d**,**i**); 400×). α-Syn is indicated by white arrows in panels showing magnified images (**e**,**j**). (**B**) The quantification of fluorescence intensity showing SGK1 and α-syn is indicated in a graph. CTL, intraperitoneally injected with 100 μL 0.9% saline daily for 4 weeks, MPTP; intraperitoneally injected with MPTP-HCI (20 mg/kg) daily for 4 weeks (*n* = 3, * *p* < 0.05, ** *p* < 0.005).

Because of the opposite expression patterns of SGK1 and α-syn (Figure 4), immunofluorescence analyses of SGK1 and α-syn were also performed in the CTL and MPTP groups to identify the correlation (Figure 5). In the MPTP group, weaker SGK1 expression (Figure 5a,f) and stronger α-syn expression (Figure 5b,g) were observed than in the CTL group. The colocation of SGK1 and α-syn was confirmed in the merged panels (Figure 5c,h), and more brightly colocated SGK1 and α-syn in the MPTP group were also identified (see white arrows in Figure 5e,j).

**Figure 6:**
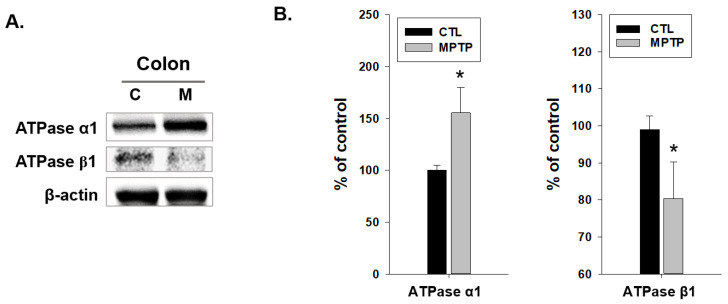
Changes in the expression pattern of Na^+^/K^+^ pump ATPase α1 and β1 in the colon in 1-methyl-4-phenyl-1,2,3,6-tetrahydropyridine (MPTP)-induced Parkinson’s disease model. (**A**) Immunoblotting analyses of Na^+^/K^+^ pump ATPase α1 and β1 expression in the colon. ATPase α1 expression increased significantly, whereas ATPase β1 expression decreased in the colon in the MPTP group compared with that in the control (CTL) group. (**B**) Graphs indicating the immunoblotting analysis results (*n* = 3, * *p* < 0.05).

It can be inferred that these morphological changes may affect the function of the colon, and water absorption may be closely related to SGK1, which is involved in the Na^+^/K^+^ pump. Thus, the factors associated with SGK1 in the Na^+^/K^+^ pump and Na^+^/K^+^ pump ATPase α1 and β1 were examined in the colons of the CTL and MPTP groups. Immunoblotting analysis indicated that ATPase α1 expression levels were significantly increased, and ATPase β1 expression levels were significantly decreased in the MPTP group (Figure 6). Immunoblot results (Figure 6A) demonstrated a significantly increased expression of ATPase α1 and a decreased expression of ATPase β1 in the MPTP group (Figure 6B).

Changed expression levels of ATPase α1 and β1 were confirmed in the MPTP-induced PD model. It was also necessary to investigate SGK1-induced changes in ATPase α1 and β1 and the correlation between SGK1 and α-syn.

### 2.4. Expressional Changes in Factors Related to SGK1 Following SW480 Knockdown by SGK1 siRNA

**Figure 7:**
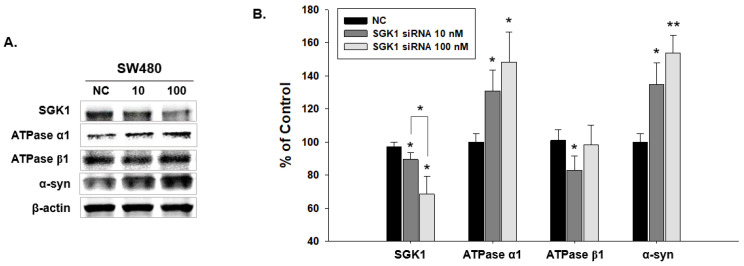
Changes in the expression pattern of serum/glucocorticoid-related kinase 1 (SGK1), α-synuclein (α-syn), and factors related to SGK1 in SW480 knocked down by SGK1 short interfering RNA (siRNA). (**A**) Immunoblotting analyses of SGK1, α-syn, and SGK1-related factors in SGK1-knockdown SW480 cells. Na^+^/K^+^ pump ATPase α1 and α-syn expression increased with decreasing SGK1 expression. The Na^+^/K^+^ pump ATPase β1 expression decreased in the 10 nM SGK1 siRNA group. (**B**) Bar graphs of the immunoblotting analysis results. NC, control siRNA treatment (100 nM for 2 days); siSGK1 10, SGK1 siRNA treatment (10 nM for 2 days); siSGK1 100, SGK1 siRNA treatment (100 nM for 2 days) (*n* = 3, * *p* < 0.05, ** *p* < 0.005).

Immunoblotting analysis was performed in SW480 cells with SGK1 knockdown to examine the changes in Na^+^/K^+^ pump ATPase α1, β1, and α-syn levels. Decreasing SGK1 levels corresponded with significant increases in the expression levels of α-syn and ATPase α1 (Figure 7). ATPase β1 decrease was significant in the SGK1 siRNA 10 group but not in the SGK1 siRNA 100 group (Figure 7B).

The results indicate that SGK1 affected α-syn and Na^+^/K^+^ pump ATPase α1 and β1 expression. Therefore, it can be inferred that SGK1 is an important factor influencing the increase in α-syn in the PD colon and affects the Na^+^/K^+^ pump system, leading to GI dysfunction associated with water absorption.

## 3. Discussion

The exact cause and mechanism of PD remain unclear. Nonetheless, the elevated levels of α-syn in the intestines are likely to play a pivotal role in the underlying pathology of PD. α-Syn in the intestine is reported to migrate to the brain and influence the pathology of PD. Therefore, increased levels of α-syn in the intestine may also increase its transport to the brain, which may affect dopaminergic neurons. Additionally, intestinal α-syn is likely to play a role in gastrointestinal diseases, which are non-motor symptoms of PD.

In the current study, α-syn expression levels were increased and SGK1 expression levels were decreased in the colon of the MPTP-induced semi-chronic PD model. As reports have highlighted, a strong association of pervasive α-syn aggregations in the gastrointestinal tract with PD [8,9,10,11] was detected, and the SGK1 expression level was decreased in the colon. SGK1 and α-syn expression patterns were found to be opposite in the surrounding colon tissue, with the expression level of SGK1 decreasing and that of α-syn increasing in the MPTP group. Immunofluorescence analyses of SGK1 and α-syn showed the colocation of SGK1 and α-syn. In the MPTP group, weaker SGK1 and stronger α-syn expression were observed compared with that in the CTL group. Furthermore, the Alcian blue staining revealed an uneven expression of mucins in the villi and microvilli of the MPTP group. These morphological changes can affect the function of the colon, and it can be inferred that water absorption could be strongly associated with SGK1, which in turn is associated with the Na^+^/K^+^ pump. SGK1 increases Na^+^/K^+^ pump ATPase cell-surface expression [32]. Thus, the factors associated with SGK1 in the Na^+^/K^+^ pump, Na^+^/K^+^ pump ATPase α1 and β1, expressed in the colon, were examined in the colons of the CTL and MPTP groups. Immunoblotting analysis indicated that Na^+^/K^+^ pump ATPase α1 expression levels were significantly increased, and those of Na^+^/K^+^ pump ATPase β1 were significantly decreased in the MPTP group compared with those in the CTL group. In SW480 cells with SGK1 knockdown, decreasing SGK1 levels corresponded with significant increases in the expression levels of α-syn and Na^+^/K^+^ pump ATPase α1. Na^+^/K^+^ pump ATPase β1 decrease was also significant in the SGK1 siRNA 10 group. These results indicate that SGK1 affected the α-syn and Na^+^/K^+^ pump ATPase α1 and β1. Na^+^/K^+^ pump ATPase is reportedly involved in water balance in the colon [33,34]. Na^+^-K^+^-ATPase α1 plays a key role in sodium water transport [35]. Na^+^/K^+^ pump ATPase β1 is required for the structural and functional maturation of Na^+^/K^+^ pump ATPase α1 [36,37]. The increase in Na^+^-K^+^-ATPase α1 induced by SGK1 reduction may reduce the need for Na^+^/K^+^ pump ATPase β1, leading to a decrease in Na^+^/K^+^ pump ATPase β1. The abnormality of these intestinal functions is one of the major non-motor symptoms of PD. Moreover, a mutation affecting Na^+^/K^+^ pump ATPase is associated with neurodegenerative disorders like early-onset PD [38,39]. Therefore, it can be deduced that SGK1, affecting intestinal α-syn and the Na^+^/K^+^ pump system, is an important factor related to the pathology of PD.

Constipation is one of the important prodromal and intestinal non-motor symptoms of PD. In addition to bowel movement, fluid and electrolyte balance is an important factor in constipation. SGK1 is a powerful stimulator of Na^+^/K^+^ pump ATPase [40,41]. SGK1-sensitive functions do not require the presence of SGK1 but are significantly regulated by SGK1 [40]. Fluid and electrolyte balance changes due to reduced SGK1 may affect the colon. Furthermore, SGK1 influences potassium exchange and balance within the colon, which can further affect fecal characteristics. These results indicate that SGK1 may affect the colon by regulating Na^+^/K^+^ pump ATPase, which is involved in maintaining electrolyte balance and fecal formation in the PD mouse model.

In a previous study, SGK1 inhibition in astrocytes and microglia ameliorated the pathology and symptoms in an animal model of PD [42]. Brain-resident glia can contribute to the progression of neuronal loss in neurodegenerative disorders by establishing a disease-harmful inflammatory environment [43]. Therefore, SGK1 inhibition in astrocytes and microglia can induce the apoptosis of these cells, thereby exerting neuroprotective effects. The injection of SGK1-expressing adenovirus into the ST of MPTP-treated mice exerted a protective effect on dopaminergic neurons [44], and the overexpression of SGK1 prevented dopaminergic cell death [23,24,25,26]. The underlying mechanism may involve the inhibition of dopaminergic cell death via the SGK1-based regulation of apoptosis [21,43]. The upregulation of SKG1, which coincides with the onset of brain dopaminergic neuron death in PD mouse models [45], may regulate apoptosis. Acute treatment with MPTP leads to a strong upregulation of SKG1 in the cytoplasmic fraction [22]. The upregulation of SGK1 strongly correlates with the occurrence of cell death [22]. This upregulation of SKG1 has been reported to play a protective role during oxidative stress conditions [22]. Downregulation studies of SKG1 showed increased cell death after oxidative stress. SKG1 forms part of the molecular pathway of cell death and seems to exert a protective role [22]. Chronic treatment with MPTP showed decreased expression of SKG1-mRNA and protein. In chronic PD, the neuroprotective mechanism involving the increased expression of SKG1 no longer operates optimally, leading to worsened neurodegeneration [28]. Moreover, SGK induces a wide range of neurotrophic effects, including the induction of neuronal hypertrophy, protection against neuronal apoptosis, and neurotoxin-induced axonal regeneration [44,46]. Therefore, reduced SGK1 levels may imply that this neuroprotective effect is no longer exhibited and that the disease may show accelerated deterioration.

In our study, a decrease in SGK1 was observed in the colon of the PD mouse model. SGK1 decrease seems to be related to the increase in intestinal α-syn, which could migrate to the brain and influence the degeneration of dopaminergic neurons. Additionally, intestinal α-syn increase is related to intestinal non-motor symptoms. Particularly, SGK1-sensitive Na^+^/K^+^ pump ATPase α1 and β1 changes are inferred to be one of the elements of intestinal dysfunction because electrolyte balance and fecal formation, which are related to constipation, could be affected by SGK1 decrease. Therefore, as currently there are no treatment or prevention methods for PD, controlling the expression level of SGK1 can be a potential approach for treating/preventing PD. To confirm this, future studies must investigate the direct relationship between SGK1 reduction and constipation in PD.

## 4. Materials and Methods

### 4.1. MPTP Model of PD

Twelve 6-week-old male C57BL/6 mice (weighting 20–22 g) were obtained from Dae Han Bio-Link Co. (Daejeon, Republic of Korea). They were divided into two groups of six mice each: CTL and MPTP group. The CTL group was intraperitoneally injected with 0.9% saline (100 μL), whereas the MPTP group was intraperitoneally injected with MPTP-HCI (20 mg/kg, Sigma, St. Louis, MO, USA) in 0.9% saline solution (100 μL) once a day for 4 weeks to develop a chronic PD model. The day after the last MPTP injection, both groups of mice were anesthetized using Alfaxan (JUROX, Braeside, Australia) and perfused with cold 0.05 M sodium phosphate buffer to perform Western blotting. The Sangji University Animal Experimentation Committee approved all the animal protocols used in this study (#2021-9).

### 4.2. Immunohistochemistry

The mice were dissected after four weeks of treatment, and the tissues were fixed using 4% paraformaldehyde for one day at 4 °C and then dehydrated in 30% sucrose buffer for three days at 4 °C. For immunohistochemical analysis, whole SN, ST, and colonic tissues were processed in a cryomicrotome and incubated in 3% H_2_O_2_ with PBS (pH 7.4). The sections were then blocked using an avidin/biotin blocking kit (Vector Laboratories, Newark, CA, USA) for 1 h and incubated with M.O.M mouse Ig-blocking reagent (Vector Laboratories, Newark, CA, USA) for 1 h at room temperature; this was followed by incubation with a primary antibody. Each section was stained with anti-TH (1:2000; Santa Cruz Biotechnology, Dallas, TX, USA), anti-α-syn (1:1000; Novus Biologicals, Littleton, CO, USA), or anti-SGK1 (1:1000; Santa Cruz Biotechnology, Dallas, TX, USA). The sections were then treated with biotinylated anti-mouse IgG and avidin–biotin–peroxidase complexes. Diaminobenzidine hydrogen peroxide solution was used to visualize the antigens. The cells in the SN corresponded to dopaminergic cells. The concentration of TH in the ST was measured using Image J (version 1.52a; NIH, Bethesda, MD, USA).

### 4.3. Alcian Blue Staining

The colon tissues were incubated in 3% acetic acid solution for 3 min and in Alcian blue solution (pH 2.5) for 30 min. The sections were dehydrated using graded ethanol. The Alcian Blue Stain Kit (H-3501, Vector Laboratories, Newark, CA, USA) was used, and recommended procedures were followed.

### 4.4. Western Blotting

For analysis, SN, ST, and colonic tissues were homogenized using 20 mM radio-immunoprecipitation assay buffer and incubated on ice for 30 min. SW480 cells were lysed using a lysis buffer. After centrifugation at 12,000× *g* rpm at 4 °C for 20 min, an equal amount of protein supernatant was separated using sodium dodecyl sulfate-polyacrylamide gel electrophoresis and then transferred onto polyvinylidene difluoride membranes (Pall Life Science, Port Washington, NY, USA). The membranes were then blocked using 3% bovine serum albumin (BSA) for 1 h and 30 min and incubated with anti-TH (1:2000), anti-SGK1 (1:2000), anti-β-actin (1:2000; Santa Cruz Biotechnology, Dallas, TX, USA), anti-Na^+^/K^+^ pump ATPase α1 (1:2000; Santa Cruz Biotechnology, USA), anti-Na^+^/K^+^ pump ATPase β1 (1:2000; Santa Cruz Biotechnology, USA), or anti-α-syn (1:500; BD Biosciences, Franklin Lakes, NJ, USA) primary antibody. The membranes were washed three times with 0.1% Tris-buffered saline (20 mM Tris-HCl (pH 7.5) and 150 mM NaCl) containing 0.1% Tween-20 and treated with goat anti-mouse IgG (1:5000; Abcam, Cambridge, UK) as a secondary antibody for 1 h. Afterward, the membranes were treated with Pierce ECL Western detection reagents (GE Healthcare, Chicago, IL, USA), and the antigen bands were visualized with an Alliance Q9 Micro chemiluminescence imaging device (UVITEC, Cambridge, UK).

### 4.5. Immunofluorescence Microscopy

Cryosectioned colon regions were washed with cold PBS and incubated in 0.3% Triton X-100 for 30 min. After incubation in blocking buffer (1% BSA, 5% goat serum in PBS) for 1 h, sections were incubated with mouse anti-SGK1 and rabbit anti-α-syn primary antibodies. After overnight incubation at 4 °C, the sections were treated with anti-mouse IgG-Alexa Fluor 488 or anti-rabbit IgG-tetramethylrhodamine secondary antibodies. Then, colonic sections were treated with 4′,6-diamidino-2-phenylindole (1 µg/mL). A Nikon X-cite series 120Q microscope (Nikon, Tokyo, Japan) was used to visualize the samples.

### 4.6. Cell Lines and Cultures

The SW480 cell line (Korean Cell Line Bank, Seoul, Republic of Korea) was cultured in Roswell Park Memorial Institute medium (WELGENE, Seoul, Republic of Korea) containing 10% fetal bovine serum (GenDEPOT, Baker, TX, USA), 100 U/mL of penicillin, and 100 mg/mL streptomycin and maintained under standard culture conditions (5% CO_2_, 37 °C).

### 4.7. siRNA Transfection

For transient knockdown, SGK1 siRNA (5′-AGG AGA ACA UCG AGC ACA ATT-3′) and negative control siRNA (5′-UUC UCC GAA CGU GUC ACG UTT-3′) were used. The siRNAs were purchased from Bioneer, Inc., Daejeon, Republic of Korea.

### 4.8. Statistical Analysis

Statistical comparisons were conducted using Student’s *t*-test for two-group comparisons and one-way analysis of variance for three-group comparisons with SPSS 25 software (SPSS Inc. Released 2017). Differences yielding *p*-values of <0.05 were considered statistically significant. All values are expressed as means ± the standard error.

## 5. Conclusions

In conclusion, the expression of α-syn increased and that of SGK1 decreased in the colon of the MPTP-induced PD mouse model. The decreased expression of SGK1 induced an increase in α-syn and changes in the expression of Na^+^/K^+^ pump ATPase α1 and β1. These results suggest that reduced SGK1 may affect the colon by regulating Na^+^/K^+^ pump ATPase α1 and β1, thereby affecting electrolyte balance and fecal formation in the PD mouse model. Gastrointestinal disorders, especially constipation, are some of the major prodromal symptoms of PD. Therefore, modulating SGK1 expression could be an important strategy to control PD. This is the first study to investigate the relationship between SGK1 and intestinal changes in a PD mouse model.

## Data Availability

All necessary data are included in the paper.

## Supplementary Materials

The following supporting information can be downloaded at: https://www.mdpi.com/article/10.3390/ijms242216408/s1.
