# Supplementary material for: Association between Decreased SGK1 and Increased Intestinal α-Synuclein in an MPTP Mouse Model of Parkinson’s Disease
Source: Int J Mol Sci. 2023 Nov 16;24(22):16408. doi: 10.3390/ijms242216408 (PMC10671719; doi:10.3390/ijms242216408)
Supplement: Supplementary file 1 [file ijms-24-16408-s001.zip › ijms-2660259-supplementary.pdf]

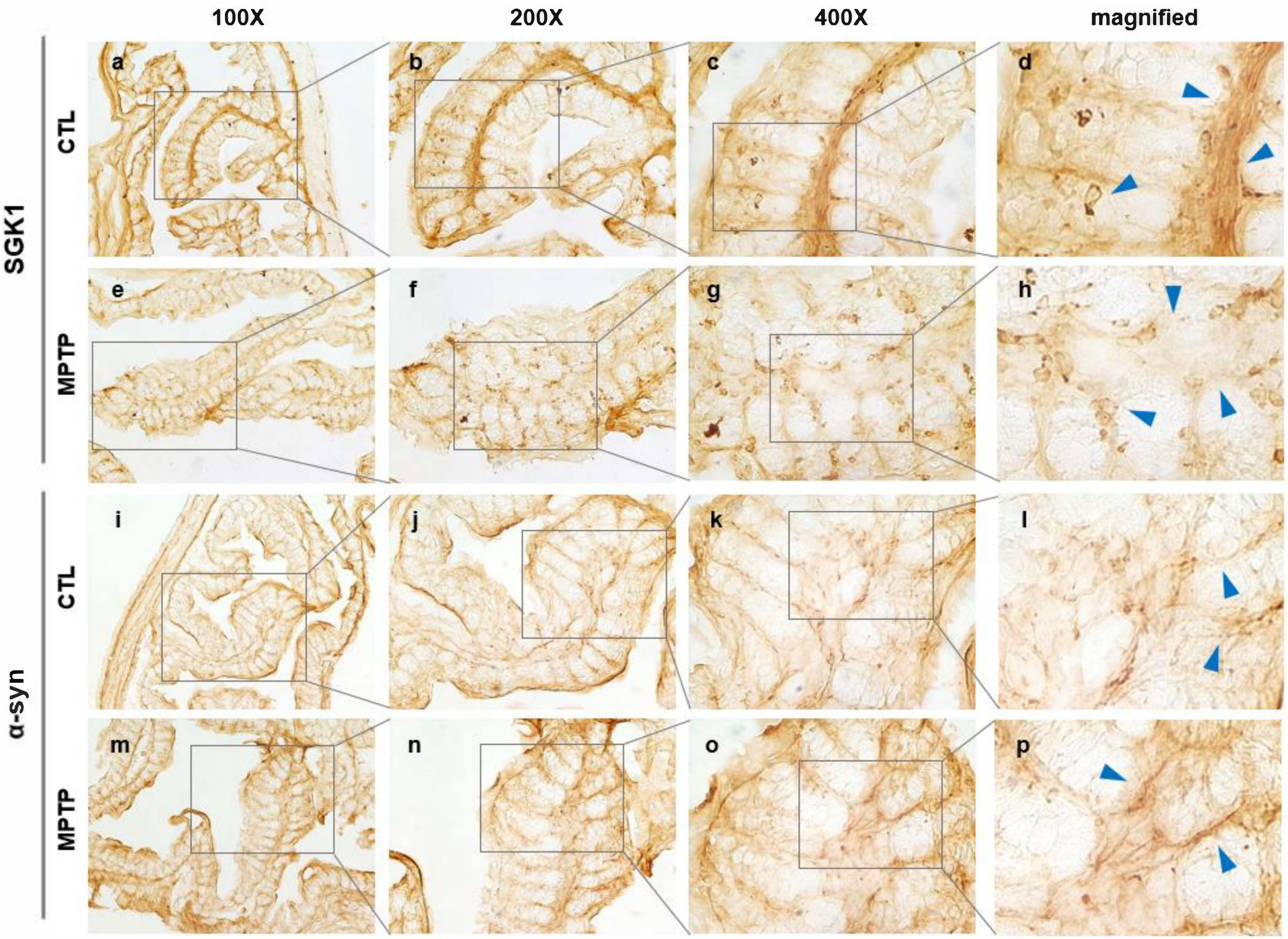

Supplementary figure S1. The serum/glucocorticoid-related kinase 1 (SGK1) and alpha-synuclein (α-syn) expression pattern changes in colon in 1-methyl-4-phenyl-1,2,3,6-tetrahydropyridine (MPTP) induced Parkinson's disease (PD) model. Immunohistochemistry analyses of SGK1 (a-h) show the decreasing SGK1 in MPTP group (e-h) comparing in control (CTL) group (a-d). On the other hand, immunohistochemistry analyses of α-syn (i-p) show the increasing α-syn in MPTP group (m-p) comparing in control (CTL) group (i-l). The SGK1 and α-syn were indicated by blue arrows in magnified panels (d,h,l and p).

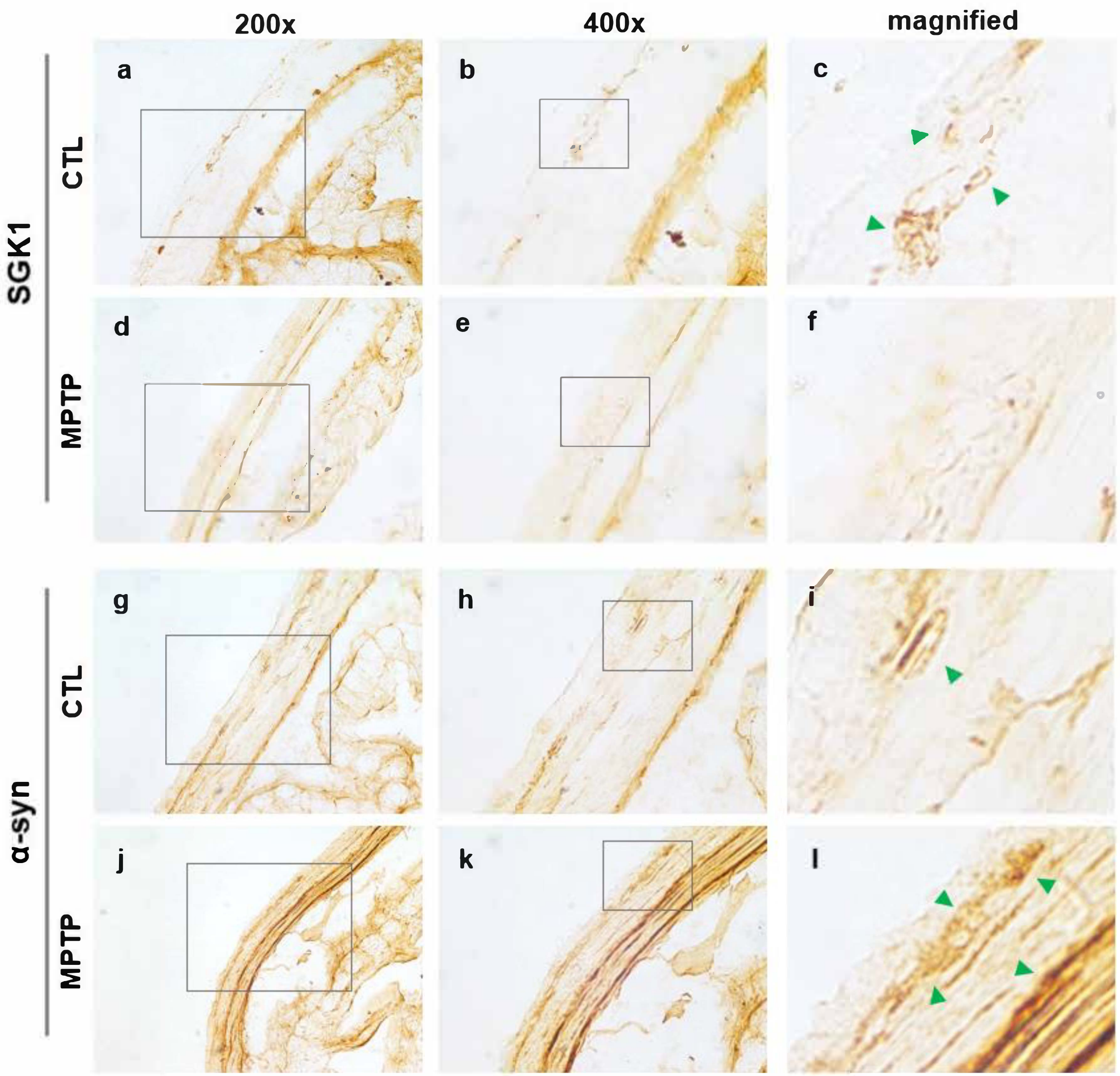

Supplementary figure S2. Changes in the expression pattern of serum/ glucocorticoid-related kinase 1 (SGK1) and α -synuclein (α-syn) in the colon in 1-methyl-4-phenyl-1,2,3,6- tetrahydropyridine (MPTP)-induced Parkinson's disease model. Immunohistochemistry analyses of SGK1 (a–f) showing decreased SGK1 expression in the MPTP group (d–f) compared with that in the control (CTL) group (a–c). Immunohistochemistry analyses of α-syn (g–i) showing increased α-syn expression in the MPTP group (m–p) compared with that in the control (CTL) group (g–i). SGK1 and α-syn are indicated by black arrows in panels showing magnified images (c, f, and l). CTL, intraperitoneally injected with 100 μL 0.9% saline daily for 4 weeks; MPTP, intraperitoneally injected with MPTP-HCl (20 mg/kg) daily for 4 weeks.
